# Neurotrophics correlates and functional remediation in bipolar disorder. A pilot study

**DOI:** 10.1192/j.eurpsy.2022.574

**Published:** 2022-09-01

**Authors:** V. Accardo, S. Barlati, A. Vita

**Affiliations:** 1 University of Brescia, Department Of Molecular And Translational Medicine, Brescia, Italy; 2 University of Brescia School of Medicine, Department Of Mental Health And Addiction Services, Asst Spedali Civili, Brescia, Italy., Brescia, Italy; 3 University of Brescia, Department Of Clinical And Experimental Sciences, Brescia, Italy

**Keywords:** Functional Remediation, bipolar disorder, NEUROTROPHICS CORRELATES, bdnf

## Abstract

**Introduction:**

Bipolar disorder (BD) is a complex mental disorder. Cognitive dysfunction represents a core feature, strongly related with patients’ functional outcome. Functional Remediation (FR), is an integrated neuropsychological and psychosocial rehabilitation treatment aimed at enhancing cognitive functions in order to achieve full functional recovery(Torrent et al., 2013). Evidence highlighted an association of neurotrophins and cognitive dysfunctions. Particularly, BDNF has been investigated a potential biomarker. Preliminary studies explored the effects induced through FR interventions on serum BDNF levels(Bonnin et al., 2019). Evidences suggest that high BDNF serum levels are related to good cognitive functioning(Mora et al., 2019). Results require further explorations. The present pilot study targets to identify the neurobiological correlates of response, investigating the potential neuroprotective role of the FR.

**Objectives:**

Assess the effectiveness of FR in ameliorate cognitive deficits measured with BAC-A and psychosocial functioning with FAST, in modifying BDNF levels in a sample of euthymic patients with BD, compared to standard treatment.

**Methods:**

Two arms(1:1)randomized, rater-blinded, controlled study of 30out-patients with BD-I and BD-II, according to DSM-5 criteria. Patients between 18 and 55 years in euthymic phase. Neurocognitive and clinical assessments, at the same times, serum assessment of BDNF levels will be performed.

All patients will be assessed at baseline(T0), at the end of treatment(T1) and at the 3-month follow-up(T2).

**Results:**

After treatment, patients receiving FR show better cognitive and psychosocial performance than those receiving TAU.

**Conclusions:**

Given the important role of neutrophins in the pathogenesis of BD, identifying BD-specific biomarkers would contribute to understand the underlying neuro-pathophysiological processes and to personalize treatments.

**Disclosure:**

No significant relationships.

